# Lysophosphatidic Acid Is an Inflammatory Lipid Exploited by Cancers for Immune Evasion *via* Mechanisms Similar and Distinct From CTLA-4 and PD-1

**DOI:** 10.3389/fimmu.2020.531910

**Published:** 2021-01-27

**Authors:** Divij Mathew, Raul M. Torres

**Affiliations:** Department of Immunology & Microbiology, University of Colorado School of Medicine, Aurora, CO, United States

**Keywords:** CD8 T cell, cancer, lysophophatidic acid, LPAR5, inhibitory receptor and ligand

## Abstract

Immunological tolerance has evolved to curtail immune responses against self-antigens and prevent autoimmunity. One mechanism that contributes to immunological tolerance is the expression of inhibitory receptors by lymphocytes that signal to dampen immune responses during the course of an infection and to prevent immune-mediated collateral damage to the host. The understanding that tumors exploit these physiological mechanisms to avoid elimination has led to remarkable, but limited, success in the treatment of cancer through the use of biologics that interfere with the ability of cancers to suppress immune function. This therapy, based on the understanding of how T lymphocytes are normally activated and suppressed, has led to the development of therapeutic blocking antibodies, referred to as immune checkpoint blockade, which either directly or indirectly promote the activation of CD8 T cells to eradicate cancer. Here, we highlight the distinct signaling mechanisms, timing and location of inhibition used by the CTLA-4 and PD-1 inhibitory receptors compared to a novel inhibitory signaling axis comprised of the bioactive lipid, lysophosphatidic acid (LPA), signaling via the LPA5 receptor expressed by CD8 T cells. Importantly, abundant evidence indicates that an LPA-LPA5 signaling axis is also exploited by diverse cancers to suppress T cell activation and function. Clearly, a thorough molecular and biochemical understanding of how diverse T cell inhibitory receptors signal to suppress T cell antigen receptor signaling and function will be important to inform the choice of which complimentary checkpoint blockade modalities might be used for a given cancer.

## Introduction

Lipid biology in the context of tumor immunity remains vastly unexplored. However its role in modulating inflammation has been used for centuries (1), which has led to pharmaceutical development of nonsteroidal anti-inflammatory drugs (NSAIDs), like COX-2 inhibitors, that inhibit the generation of prostaglandin and thromboxane lipids. Interestingly, COX-2 inhibitors also lower cancer promoting inflammation and drive type I immunity, demonstrating a unique role of lipids affecting the anti-tumor response (2). This review seeks to highlight another bioactive lipid, lysophosphatidic acid (LPA), and its role in dampening tumor immunity. Unlike COX-2, LPA suppresses the anti-tumor response in a cell intrinsic manner by signaling via an LPA receptor (LPAR) similar to checkpoint receptors. Furthermore, as LPA is found systemically, and in all tissues, we speculate mechanisms of suppression mediated by LPARs are similar to CTLA and PD-1 in inhibiting T cell antigen receptor (TCR) signaling, yet via distinct signaling pathways. Specifically, LPA signaling has been shown to suppress T cell TCR Ca^2+^ signaling which inhibits naïve T cell activation in secondary lymphoid organs. Additionally, as tumors can produce LPA at higher concentrations than adjacent tissue, this tumor-derived LPA also inhibits T cell effector function therefore representing a checkpoint in T cell function similar to that mediated by CTLA-4 and PD-1. Therefore, as a lipid, LPA/LPAR modulation of immune responses has functional similarities to other checkpoint molecules like PD-1/PD-L1 or CTLA-4/CD80 yet remains unique to other soluble or cell-associated protein-protein interactions.

## CTLA-4: Suppression of Early T Cell Activation

CTLA-4 is a major inhibitory receptor expressed by CD8 T cells and was an initial therapeutic target given that CTLA-4 is expressed following TCR engagement by naïve T cells and continues to rise until maximum expression at 48 h (3). Compared to the CD28 T cell costimulatory receptor, CTLA-4 displays significantly higher affinity for the CD80 and CD86 co-receptors expressed by antigen presenting cells (APCs). Thus, when expressed, CTLA-4 effectively sequesters CD80 and CD86 away from CD28 and prevents co-stimulatory signaling activity for the T cell antigen receptor (TCR) (4). Furthermore, CTLA-4 interaction with CD80/CD86 can also lead to transendocytosis, the physical capture of these ligands and removal from the surface of APCs, thereby limiting total levels of available ligands for CD28 and subsequent co-stimulation (4). The physical sequestration of CD28 ligands ultimately prevents optimal TCR signaling by CD8 T cells and is an important mechanism by which CTLA-4 suppresses T cell function (5). This process of CTLA-4-mediated CD80/CD86 transendocytosis is seen in all T cell subsets but particularly in regulatory T cells contributing to the suppressive abilities of this T cell subpopulation (5). Notably, deletion of the CTLA-4 cytoplasmic tail has been reported not to change its ability to suppress T cell proliferation indicating CTLA-4 intracellular signaling is not necessary for all T cell inhibition (6). Furthermore, agonist signaling via CTLA-4 does not induce significant changes in T cell gene expression also suggesting a mechanism of inhibition that does not rely on intracellular signaling (7). However, coimmunoprecipitation of CTLA-4 in a T cell hybridoma reveals interaction with PKC-η (8). Tregs depleted of PKC-η have compromised suppressive function both *in vitro* and *in vivo* thus suggesting a cell-intrinsic signaling role for CTLA-4 (8). The significance of immune inhibition provided by CTLA-4 is highlighted by the fact that *Ctla4*^–/–^ mice survive only for the first 3–4 weeks and then die as a result of massive lymphocytic infiltration and destruction of major organs (9–11). Thus, a CTLA-4-deficiency leads to uncontrolled expansion of CD4 T cells, including autoreactive T cells that subsequently damage host organs thus indicating that CTLA-4 is required for appropriate maintenance of peripheral tolerance.

Under normal conditions, T cells that develop in the thymus and recognize self-antigens with relatively high affinity are culled from development by mechanisms of central tolerance. Given that all tumors originate from normal cells, the (non-mutated) tumor antigens they express are essentially self-antigens and central tolerance similarly deletes host CD8 T cells able to recognize tumor antigens with high affinity. Nevertheless, central tolerance is not absolute and does not remove all self-reactive T cells, especially those that display weak affinity for self-antigens. These weakly autoreactive T lymphocytes emigrate to the periphery and are further restrained by mechanisms of peripheral tolerance. Indeed, early experiments designed to block the CTLA-4 inhibitory receptor to improve the endogenous self/tumor host response against a murine 51BLim10 colon cancer resulted in immediate rejection of the tumor (12). Accordingly, anti-CTLA-4 blockade therapy has shown success in the clinic, however, this approach also presents with treatment related toxicities such as nausea and fatigue seen in 70–80% of patients or dermatitis and enterocolitis seen in 5–25% of patients (13). Recently, anti-CTLA-4 antibodies have been engineered to behave differently during the endosomal trafficking of CTLA-4 to minimize these adverse events. Specifically, ipilimumab harboring tyrosine-to-histidine mutations display increased pH sensitivity and upon entering the endosomes bound to CTLA-4, disengages from CTLA-4 in the lysosomes allowing CTLA-4 to recycle to the surface in a LRBA dependent manner which still induces tumor regression with minimal associated adverse events (14). Of note, recent data has suggested that an alternate mechanism by which anti-CTLA-4 antibodies act is via the depletion of regulatory T cells (15, 16), thus questioning its mechanism of action as a checkpoint blockade therapy and suggesting it may instead act as an antibody depleting therapy. In contrast, while treatment of patients with either ipilimumab or tremelimumab anti-CTLA-4 monoclonal antibodies has led to increased (CD4 and CD8) T cell tumor infiltration, the number of tumor-infiltrating FOXP3+ regulatory T cells was not significantly altered (17). Thus, blocking CTLA-4 represents a form of checkpoint blockade that allows for greater primary expansion of effector T cells; however, the precise mechanism by which CTLA-4 modulates CD8 T cells in the tumor microenvironment is less certain.

## PD-1: Co-Opted by the Tumor Microenvironment

As CTLA-4 expression is initiated soon after initial T cell antigen-recognition by naïve T cells, its mechanism of T cell suppression is considered to be primarily restricted to secondary lymphoid organs where T cells typically first encounter foreign antigens. However, additional inhibitory receptors are also expressed by CD8 T cells and able to suppress cytotoxic function at various stages. PD-1 is another inhibitory receptor expressed by activated CD8 T cells and able to dampen effector function and has received abundant attention as a target of immune checkpoint blockade. Unlike CTLA-4, PD-1 harbors both ITIM and ITSM tyrosine-based motif sequences in the cytoplasmic domain (18) that facilitate inhibitory function and arguing for a mechanism of suppression dependent on receptor signaling. While it is generally considered that inhibition mediated by PD-1 is promoted through the recruitment of cytosolic phosphatases, it remains unclear precisely which stimulatory signals are the targets of inhibition. Immunoprecipitation of CD3ζ shows a ~70% reduction of phosphotyrosine when TCR and PD-1 are co-ligated in comparison to TCR ligation alone (19). Furthermore, both SHP-1 and SHP-2 are thought to mediate this suppression as both were found to immunoprecipitate with the ITSM domain of PD-1 (17). However, as PD-1 preferentially clusters with CD28 rather than the TCR, this argues that CD28 is the preferential target of PD-1 signaling (20). In fact, using a cell-free FRET-based assay, it was determined that PD-1 selectively recruited SHP-2 which in turn dephosphorylated CD28.

Despite discrepancies in the described mechanism(s) of PD-1 inhibitory action, PD-1 blockade has enjoyed major success in the clinic. This is because a major ligand for PD-1 is PD-L1, whose expression is upregulated by diverse tumors in response to IFNγ. In fact when examined, ~98% of PD-L1 expressing melanocytes were co-localized with T cells as opposed to minimal co-localization of T cells with PD-L1-negative tumor cells, suggesting tumor cells express PD-L1 in response to infiltrating T cells (21). Thus, attenuating this inhibitory signal prevents T cell suppression and leads to increased cytotoxicity from tumor-specific CD8 T cells. Indeed, the success of anti-PD-1/PD-L1 signaling is highlighted by having garnered FDA approval for the treatment of kidney cancers, melanomas, prostate cancers, lung cancers, B cells lymphomas, Hodgkin’s lymphoma, urothelial carcinomas, gastric cancers, liver cancers, cervical cancers, and head and neck cancers in the last 5 years (22). Although the majority of evidence suggests the PD-1/PD-L1 signaling axis acts primarily at sites of chronic inflammation, recent data provides evidence for a role for PD-1 signaling during the early phases of T cell activation (23). Specifically, upon TCR-mediated activation, PD-1 expression by CD8 T cells is upregulated within 4 h, matching the kinetics of the CD25 activation antigen and preceding cell division, thus arguing for a physiological role for PD-1 during primary activation (23). PD-L1 blockade on day 0 and day 3 after LCMV Armstrong infection led to increased granzyme B and mTOR signaling two days later by CD8 T cells (23). Thus, PD-1 signaling appears to suppress T cell function at various stages representing another ‘checkpoint’ that tumors exploit to escape elimination.

Given the clinical success and limitations of these therapeutic interventions for cancer, additional effort is needed to better understand precisely how T cell function is regulated by both stimulatory and regulatory signals (24). Ongoing and new research has identified novel protein inhibitory receptors and below we further describe a lipid that signals via a cognate G-protein coupled receptor (GPCR) to deliver suppressive signals to CD8 T cells and which ultimately negatively-regulate T cell function.

## Lysophosphatidic Acid

Lysophosphatidic acid (LPA) is a lysophospholipid structurally similar to sphingosine-1-phosphate (S1P), a lipid that has been well characterized to signal to immune cells and to orchestrate cell trafficking (25). Both lipids share a phosphate head group attached to a glycerol backbone; however, LPA differs by having a single ester linked aliphatic chain whereas S1P has a single amine linked aliphatic chain. On initial discovery both LPA and S1P were considered to be intracellular lipid metabolites and only later were characterized to function as extracellular bioactive lipids that signal to cells expressing cognate G-protein coupled receptors (GPCRs). Extracellular LPA is generated predominantly via the enzymatic activity of Autotaxin (ATX), a secreted ectoenzyme with lysophospholipase D activity that hydrolyzes the abundantly available lysophosphatidylcholine to produce LPA. Although five isoforms of ATX exist through alternative splicing of exons 12, 19, and 21, ATXβ is the form most expressed in tissue (26). Autotaxin is encoded by *ENPP2* and is highly expressed in nervous system as well as considerable expression by stromal and endothelial cells with reduced general expression in most other tissues. Structural studies have indicated that Autotaxin harbors an exposed integrin binding motif and, as a secreted enzyme, Autotoxin is thought to associate with surface-bound integrins (27, 28). Thus, current models posit that in certain microenvironments integrin-bound Autotaxin hydrolyzes lysophosphatidylcholine to produce LPA where localized concentrations are able to signal via LPARs expressed by nearby cells, including cell types not producing the Autotaxin enzyme. Extracellular LPA production also appears tightly regulated with a half-life of approximately three minutes due to its rapid hydrolysis mediated by Lipid phosphate phosphatases (LPP) 1 and LPP3. The half-life of LPA increases 4 fold when intravenously introduced into mice deficient for LPP1 (lipid phosphate phosphohydrolase type 1), an enzyme that degrades LPA, and *Lpp1*^–/–^ mice harbor higher levels of LPA (29).

**Figure 1:**
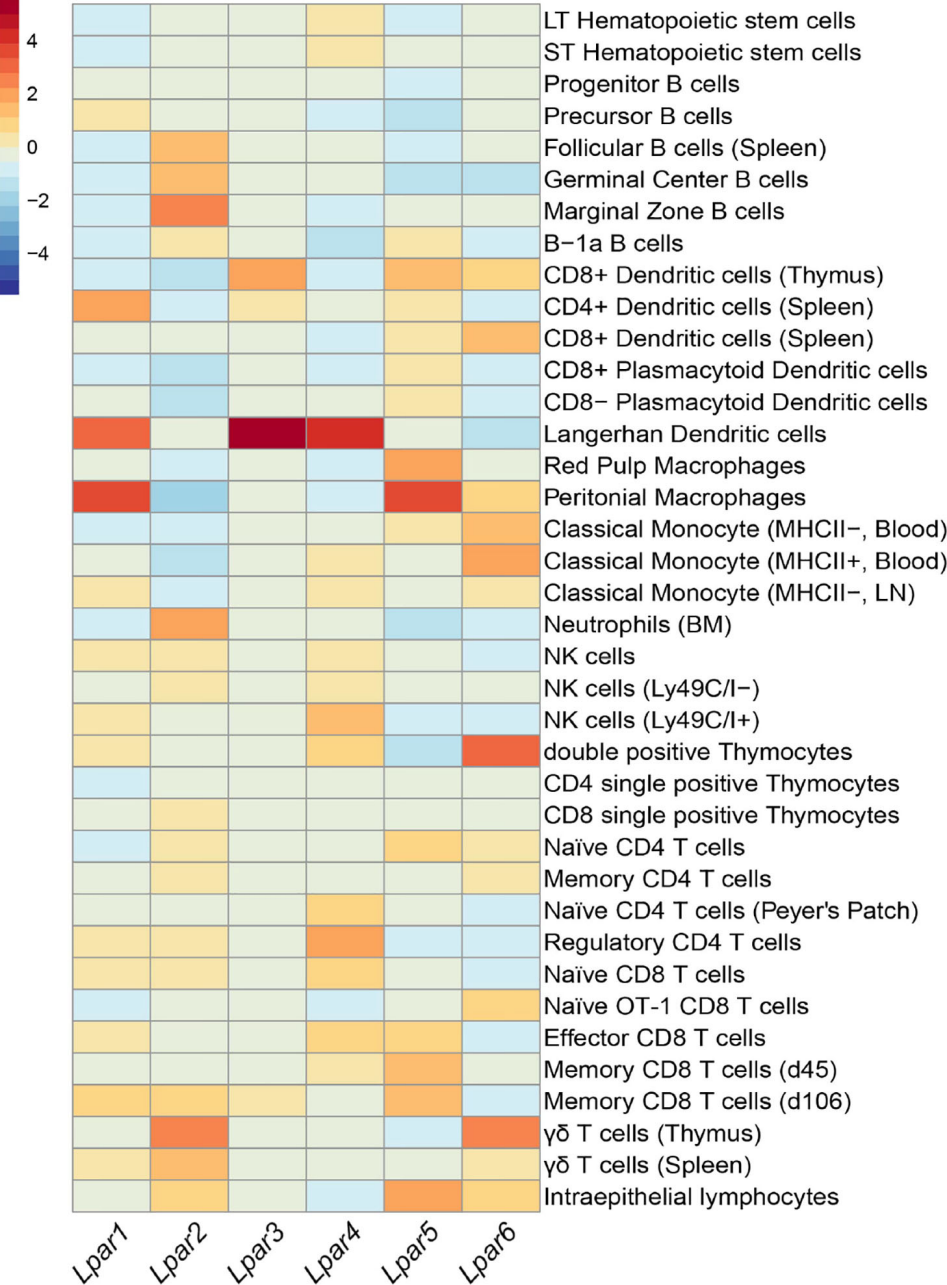
Summary of *Lpar* expression in different leukocytes. Heatmap showing expression of LPA receptors across immune subsets. Data was compiled from Immune Genome Project microarray of sorted immune populations and scaled to columns (30). Color scale on the top left indicates level of mRNA expression. Unless specified, immune populations were sorted from 6-8 week C57BL/6 mice.

Similar to S1P, LPA also signals via cognate GPCR receptors of which 6 LPA receptors (LPA_1–6_) have been characterized and that are variably expressed on all immune populations (**Figure 1**). Thus, given its ability to signal extracellularly and act as an intracellular second messenger (31, 32), it is not surprising that LPA has been associated with a number of physiological processes including smooth muscle contraction, platelet aggregation, and blood pressure (33–35). Systemic changes in LPA levels have been observed in pregnancy (36), aging (37), and between sexes where females have been reported to harbor significantly elevated LPA serum levels compared with males (37). Interestingly, all of these circumstances can be considered to require suppression of inflammation. However, its role in wound repair (38) would speak directly to the suppressive affect LPA signaling has on CD8 T cell function.

**Figure 2:**
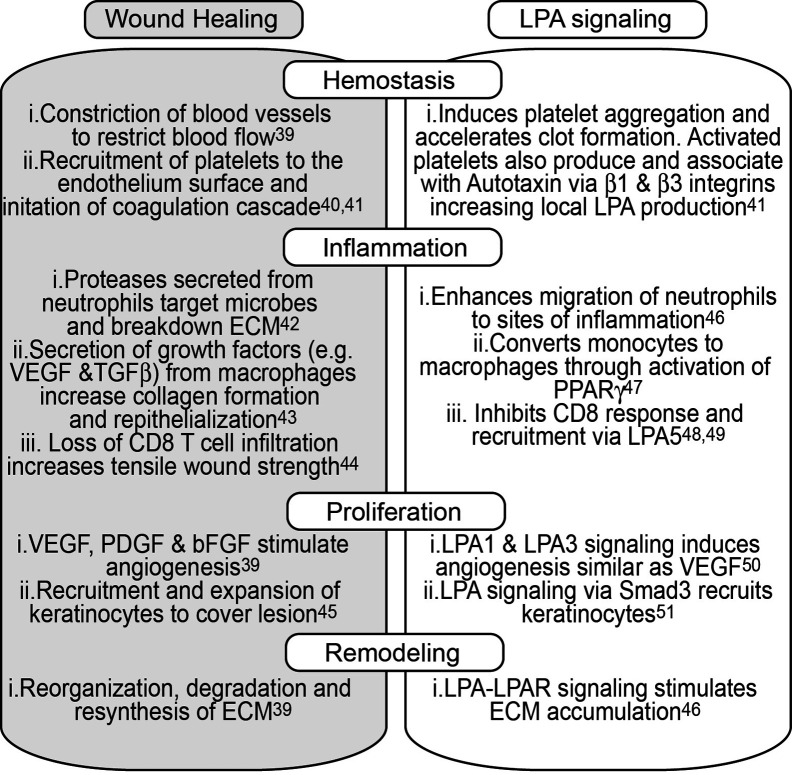
The physiological role of LPA signaling in wound repair. A simplified version of the major biological processes that occur during each step of wound healing stratified across four major groups; Hemostasis, Inflammation, Proliferation, and Remodeling. Each group is further broken down to biological events that characterize each group (grey tile) and the role of LPA in each of those biological events (clear tile).

Resolution of a wound can be subdivided into 4 distinct phases: hemostasis, inflammation, proliferation, and remodeling (39) (**Figure 2**). Immediately after a physical trauma, platelets are first to arrive to help initiate the coagulation cascade and help activate fibroblasts and recruit both neutrophils and macrophages through the secretion of TGFβ (40). Interestingly, activated platelets are a potent source of LPA and soluble Autotaxin can associate with platelet integrins and produce LPA (41). Neutrophils are the initial immune cells to infiltrate wounds and help drive an inflammatory response to eliminate any microbes (42). Although short-lived cells, the infiltration of neutrophils is vital for the production of various growth factors like IL-17 and VEGF which help in the proliferation of fibroblasts, keratinocytes, and endothelial cells. Loss of early neutrophil recruitment delays epithelialization and decreases neovascularization at the site of injury (43). While LPA does not appear to have any direct effect on neutrophil migration per se, it is able to enhance the migratory response of neutrophils to suboptimal concentrations of N-formyl-L-methionyl-L-leucyl-phenylalanine (fMLP) (46) suggesting a role for LPA in aiding neutrophil migration to sites of inflammation. Monocyte recruitment, and subsequent differentiation into macrophages, occurs 5 to 6 h post injury. Anti- inflammatory macrophages are involved with the secretion of TGFβ, clearing cellular debris, helping reorganize the extracellular matrix (ECM) and contracting the wound. Macrophage-mediated degradation of the ECM leads to more endothelial proliferation and the release of angiogenesis factors such as FGF and placental growth factor (PIGF) (52). Of note, LPA is able to directly promote the conversion of monocytes to macrophages and is true in both humans and mice (47). In fact, culturing monocytes in media containing only LPA converts CD11b^+^F480^-^ monocytes into CD11b^+^F480^+^ macrophages more so than only M-CSF (47). While the role of T cells in wound repair remains largely unexplored, depletion of CD8 T cells increases tensile strength across lesions suggesting some inhibitory role of CD8 T cells (44). Given this overlap of LPA-mediated effects and wound healing, it is not surprising that topical application of LPA to physical wounds in rats or mice promoted accelerated healing with increased neoepithelial thickness (53, 54), an effect further seen in aged rats compared to young rats (55). As the recruitment and proliferation of keratinocytes remains critical for skin repair, LPA signaling not only induced increased migration and expansion of keratinocytes but also induced a four to eight fold increase in TGFα production (45).

## LPA as a Cancer Intrinsic Growth Factor

Tumors have been appreciated to rely on pathways used in wound repair and have often been described as wounds that never heal (56). For example, IL-1β, IL-6, and IL-8 are cytokines that are secreted during the early inflammatory response after tissue damage and are involved with re-epithelization. However, in breast cancer, these cytokines are associated with a poor prognosis as they have been linked to tumor growth and metastasis (57–59). Similarly, LPA has been exploited by cancers to promote growth in several non-redundant ways. LPA signaling has directly been linked to hTERT upregulation as ovarian cancer lines treated with LPA have increased telomerase activity as early as 12 h after co-culture (60). This replicative advantage mediated by LPA helps explain the increased expression of the gene encoding Autotaxin, *ENPP2*, observed in ovarian cancer stem cells; a population of long lasting malignant cells that seeds cancer growth (61). In fact, autocrine LPA signaling in these cells has been shown to promote sphere forming ability and upregulation of ALDH, markers associated with cancer stem cells. This dependence on LPA signaling by ovarian cancer cells provided rationale to explore the use of LPA as a potential biomarker in ovarian cancer progression and accounts for the high levels of LPA found in ascites fluid from individuals with ovarian cancer (62). LPA signaling can also directly affect several ‘hallmarks’ of cancer (63), including proliferation (64), or metastasis of colorectal cancers (65), increased angiogenesis in transformed NIH3T3 cells (66) and further demonstrated in chorio-allantoic membrane assays (50), resisting cell death by increasing insensitivity to chemotherapy in ovarian cancers (67), altered lipogenesis as LPA increases fatty acid synthase in ovarian cancers (68), and by modulating inflammation through activation of PPAR-γ in the tumor stroma (69).

## LPAR SIGNALING as a ‘Checkpoint’ in the Anti-Tumor Response

Given the mechanisms by which CTLA-4 and PD-1 suppress T cell function, the LPA signaling axis can be considered as an additional form of suppression. However unlike CTLA-4, which inhibits activating signals of antigen presenting cells to CD8 T cells, or PD-1, which inhibits interactions of effector T cells and targets cells, we find LPA signaling disrupts T cell engagement with APCs and target cells (48, 49). In initial work from our lab, we demonstrated that LPA engagement with the LPA5 receptor induces a signal that inhibits TCR-induced Ca^2+^ release from intracellular stores in naïve CD8 T cells (48). This suppressive LPA signaling is dependent on the LPA5 receptor, as intracellular Ca^2+^ levels are not depressed in LPA5-deficient CD8 T cells after TCR signaling is induced in the presence of LPA (48, 49). Evidence in B lymphocytes strongly suggests LPA inhibition of antigen receptor signaling manifests via impaired IP_3_ receptor activity, thereby limiting the amount of Ca^2+^ released from ER stores after antigen receptor stimulation (70). Of note, the activity of antigen receptor proximal signaling molecules, e.g., tyrosine kinases and PLCγ, are unchanged in the presence of LPA (70). Thus, the mechanism of inhibition imposed by LPA5 on CD8 T cells differs from how PD-1 and CTLA-4 suppress CD8 T cells. We have documented that LPA5 signals via Gα_12/13_-assoicated heterotrimeric G-proteins in lymphocytes and subsequently relies on the ARHGEF1 intracellular signaling effector molecule for antigen receptor mediated suppression of LPA (70). This is in contrast to CTLA-4 which can inhibit T cell function in the absence of intracellular signaling and PD-1 which depends on the recruitment of SHP-1 or SHP-2 for its suppressive action. Given the pleiotropic downstream effects of Ca^2+^ dependent processes that result from TCR signaling, it is perhaps not surprising that TCR-stimulated CD8 T cells fail to appropriately activate and proliferate in the presence of LPA both *in vitro* and *in vivo* (48). Moreover, *in vivo* LPA-induced suppression was not observed with CD8 T cells harboring null *Lpar5* alleles (48). Importantly, we have more recently determined that in addition to suppressing TCR-induced cytosolic calcium mobilization, LPA also inhibits TCR-driven ERK activation (49) and both calcium and ERK have been previously shown to be required for granule exocytosis (71–74). Accordingly, in the presence of an LPA5 agonist, effector CD8 T cells display impaired perforin localization to the immunological synapse upon cognate antigen stimulation (49). These data demonstrate LPA engagement of LPA5 is able to suppress cells at different stages of CD8 T cell maturation and characteristic of other checkpoint regulators. As a consequence, *Lpar5^–/–^* tumor-specific CD8 T cells are able to provide better control of tumor burden 8 days after adoptive transfer compared with wild type tumor-specific CD8 T cells (48, 49).

Given the negative regulation of CD8 T cells by an LPA-LPA5 axis, one might expect that a deficiency in LPA5 receptor expression or a reduction in systemic levels of LPA might lead to autoimmunity, as observed with CTLA-4-deficient mice (7–9). Interestingly, neither the *Lpar5^–/–^* nor *Enpp2^+/–^* (ATX heterozygous) mice, which harbor half the normal levels of systemic LPA, appear to present with any obvious systemic inflammatory conditions, raising questions about the role of LPA as a suppressive lipid. However, we note that PD-1-deficient mice develop significantly less severe immune pathologies compared to *Ctla4^–/–^* mice and neither the Tigit*^–/–^* nor CD96*^–/–^* mice present with spontaneous disease (75).

Together, these findings highlight the different functions displayed by inhibitory receptors. Furthermore, as postulated by the ‘tide model’ (76), the existence of multiple costimulatory and coinhibitory receptors on T cells suggest that T cell signaling is finely tuned and responds to the microenvironmental context in which TCR signaling occurs. Thus, while certain signals appear more paramount (e.g., CD28, CTLA-4, PD-1), certain contexts reveal the dominance of some (inhibitory) receptor signaling over other signals, as evident by the greater expansion of autologous CD8 T cells with DCs with anti-LAG3 blockade over anti-PD-1 blockade (77). Given that a majority of monotherapy checkpoint blockade fails to induce tumor remission, we propose that inhibition of the LPA signaling axis represents another potential ‘checkpoint’ to target in combinational therapy.

**Figure 3:**
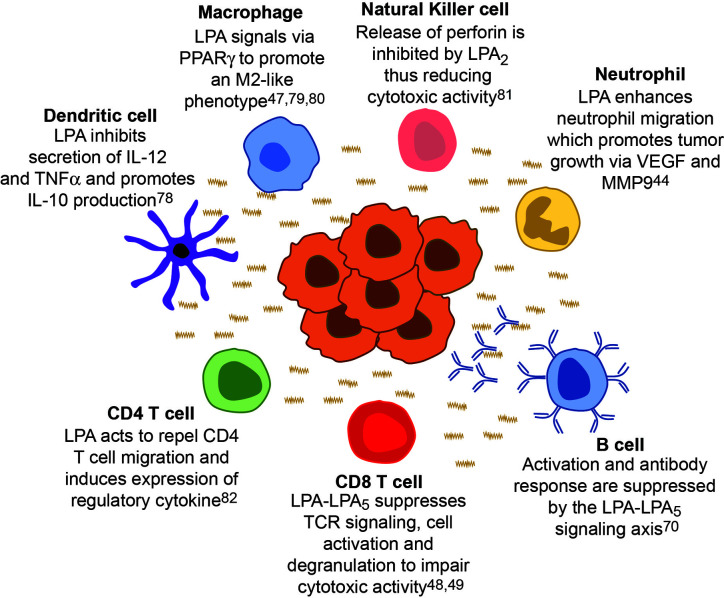
Cancer derived LPA regulates immunity across all leukocytes. LPA (wavy line) originates from the tumor (center cellular mass) and suppresses the function of all leukocytes within the microenvironment creating a ‘checkpoint’ in the immune response against cancer. Each major leukocytes population is around the tumor mass with a specific example of how LPA signaling affects that immune cell, thereby generating a tumor promoting environment.

As all immune cells express at least one LPA receptor, it is reasonable to consider that this bioactive lipid has a role in modulating antitumor function in other tumor infiltrating leukocytes. (**Figure 3**) (78–80, 83). In fact, the suppression mediated by LPA signaling can extend beyond CD8 T cells to other cells in the adaptive arm. LPA can impair the migration of CD4 T cells and even causes chemorepulsion *in vitro* in a LPA2-dependent manner (82). Moreover, in the presence of LPA, stimulated human CD4 T cells can produce IL-13, a Th2 cytokine involved with the activation of myeloid derived suppressor cells; thus, the reduced CD4 T cells that do migrate to the tumor are still involved in maintaining a pro-tumor environment (84, 85). Unlike CD4 T cells, LPA can act as a chemoattractant to natural killer (NK) cells yet also impair effector function. LPA signaling though LPA2 can increase cAMP levels and activated protein kinase A which subsequently inhibits the release of perforin in NK cells (81). Inhibition of protein kinase A activation restores NK cell cytotoxicity in the presence of LPA, suggesting a mechanism by which tumor derived LPA can impair NK function. Similar to the of CD8 T cells, B cells signaling and function are also inhibited through LPA5 (70). Specifically, LPA signaling though an LPA5–Gα_12/13_–Arhgef1 axis results in reduced Ca^2+^ signaling, a mechanism similar to CD8 T cells. Functionally, LPA signaling reduced humoral responses to T1-2 antigens suggesting a conserved inhibitory signal in T and B cells mediated through LPA5 (70). Functional changes mediated through LPA signaling extends further than lymphocytes as dendritic cells, macrophages, and neutrophils are altered in the presence of this lipid. While LPA signaling does not affect endocytic function of dendritic cells, it does inhibit the secretion of IL-12 and TNFα while promoting the secretion of IL-10 (78). Thus, dendritic cells in the tumor microenvironment exposed to LPA would be poised for a tumor promoting function. Unlike CD4 T cells, LPA is a chemoattractant to neutrophils (46, 82). However this potenial influx of neutrophils has been linked to reduced overall survival in several cancers, presumably due to release of tumor promoting factors like VEGF or MMP9 (89). Futhermore, tumor infilitrating neutrophils can secrete TGFβ and have been implicated to changing the plasticity of macrophages to an M2-like state which promotes tumor growth (89). As LPA signaling though PPAR-γ can cause the differentiation of monocytes to macrophages, we speculate the LPA signaling is involved with the influx and differentiation of tumor promoting myeloid cells (47, 79, 80).

We believe LPA signaling has evolved to help with would repair to prevent an overactive immune response. Consistent with this, cancers exploit this very system to suppress immune cell function thereby representing a “checkpoint” in our endogenous antitumor response. We propose that manipulation of the LPA/LPAR axis should be considered for potentially synergizing with anti-PD1, anti-CTLA4 or other therapies to improve leukocyte function in the tumor microenvironment (51).

## Author Contributions

DM and RT wrote and edited the review. All authors contributed to the article and approved the submitted version.

## Funding

This work was funded by NIH/NIAID R01AI143261(RT), T32AI007405 (DM) and the Cancer League of Colorado (RT).

## Conflict of Interest

The authors declare that the research was conducted in the absence of any commercial or financial relationships that could be construed as a potential conflict of interest.
